# Construction of pectinase gene engineering microbe and its application in tobacco sheets

**DOI:** 10.1515/biol-2025-1185

**Published:** 2025-10-18

**Authors:** Jiafeng Bai, Xiaoqin Peng, Yi Zhou, Zhizhong Hu, Lili Qu, Changtong Lu, Chunping Xu

**Affiliations:** Technical Center of China Tobacco Guangxi Industrial Co. Ltd, Nanning, 530001, People’s Republic of China; Technical Center of Guangxi Zhenlong Industrial Co., Ltd, Zhongshan, 542600, People’s Republic of China; Technical Center of China Tobacco Henan Industrial Co. Ltd, Zhengzhou, 450016, People’s Republic of China; College of Food and Biological Engineering, Zhengzhou University of Light Industry, Zhengzhou, 450002, Henan, People’s Republic of China

**Keywords:** pectin degradation, genetic engineering, pectinase, tobacco sheet

## Abstract

Pectin has bad effects on the sensory quality of cigarettes. In order to reduce the pectin content in tobacco leaves, polygalacturonase (PG) gene was extracted from *Aspergillus niger* sw06, and recombinant plasmid pPICZαA was constructed and transformed into *Pichia pastoris* X33 to build an engineered strain X33/pPICZαA-PG. Transformant genomic fragment was 1,608 bp. The genomic fragment was amplified and recovered, and sequencing indicated that PG gene expression have been successfully inserted into *P. pastoris* expression vector. Positive clones were detected by SDS protein with a molecular weight of about 60 kDa. The enzyme production cycle of the recombinant strain was 36 h, and crude enzyme activity was 2872.91 U/mL. The fusion protein was purified by nickel Sepharose affinity chromatography. A clear band was detected and the concentration of recombinant protein was 8.1 μg/μL. It showed a good effect on degrading pectin after addition of the PG crude enzyme produced by recombinant yeast on the tobacco pulp. The optimized addition amount on process product line was 0.8%, which could reduce tobacco pulp pectin from 3.65 to 3.01% and achieve a degradation rate of 17.53%. Sensory evaluation showed that the effect was better when the addition amount of pulping was 0.4%.

## 1 Introduction

Pectin, a major structural component of plant cell walls, including tobacco [1], is a polysaccharide primarily composed of α-1,4-linked galacturonic acid residues [2]. During cigarette combustion, pectin affects the sensory quality and produces methanol that can be oxidized into formaldehyde and formic acid. In addition, 1–1.5% acetic acid is generated by pectin in fermentation process, which increases the spiciness and irritation of smoke and stimulate choking [3]. Furthermore, due to its hydrophilic nature, pectin exhibits strong hygroscopicity, adversely affecting tobacco combustibility. Elevated pectin levels in cigarette products have been correlated with increased tar generation [4]. Therefore, pectin is a negative factor for smoking safety.

With the development of biotechnology, the study on pectinase to degrade pectin in tobacco is increasing year by year [5]. Pectinase is a general term for a class of enzymes capable of degrading pectin molecules, with various types including polygalacturonase (PG), pectinesterase and pectinate lyase [6]. Sources of pectinase include animals, plants, and microorganisms, among which microorganisms are widely applied because of their wide variety, high yields, and high efficiency in the production of pectinase [7]. Research on the application of pectinase in tobacco processing has made significant progress in recent years, primarily focusing on efficient pectin degradation to improve tobacco quality, reduce the formation of harmful substances, and develop new enzymatic hydrolysis technologies. A penicillin-based method was used to degrade pectin content in reconstituted tobacco leaves, achieving pectinase activity as high as 3,000 U/mL and a pectin degradation rate of 28.6%. Subsequent evaluation of the treated tobacco samples for cigarette applications showed enhanced overall sensory quality, although a slight reduction in strength was observed [8]. Fan et al. [9] studied the changes in volatile aromatic components of tobacco stems after pectinase treatment, finding that the degradation products of carotenoids, phenylalanine, and neopentadiene in tobacco stems were increased to varying degrees, while the Maillard reaction products and phenylalanine degradation products also showed a certain degree of increase. Dai et al. [10] isolated high-yield pectinase-producing microorganisms from the surface of high-quality flue-cured tobacco leaves and applied them to inferior tobacco leaves to improve their usability. Yu et al. [11] sprayed a solution of pectinase onto tobacco leaves, effectively reducing the pectin content in the leaves and increasing the total amount of neutral aromatic substances.

In particular, fungi such as *Penicillius* sp., *Aspergillus* sp., *Mucor* sp., and *Rhizopus* sp. have high yields of pectinase [12]. However, natural strains often exhibit inherent limitations, including insufficient enzymatic activity and limited productivity. To address these challenges, physical, chemical, and genetic engineering approaches have been employed to develop high-performance industrial pectinase-producing strains [13]. The structural gene pelA of alkaline pectinase of *Bacillus licheniformis* DG-3 was obtained and amplified by PCR, which was successfully expressed in *Escherichia coli* [13]. An engineering strain *E. coli* BL21(pET29a-pelA) producing alkaline pectinate lyase was constructed. The endogenous poly arabinose AbnC gene was cloned from the genome of strain *Penicillium* sp. Y702 by overlapping PCR, and expressed it in *Pichia Pastoris* GS115, which increased the AbnC enzyme activity threefold [14]. The PG gene contained in *Aspergillus oryzae* PO strain was cloned and expressed it in *Pichia pastoris* GS115, which expressed enzyme activity 558 times higher than that of wild bacteria [15]. Genetically engineered strains (such as *Pichia pastoris*) have advantages such as short fermentation cycles and easy purification of enzyme products. Compared with the fermentation processes of natural fungi such as *Penicillium*, they are easier to scale up and can reduce the amount and cost of enzyme preparations in industrial production [16]. At the same time, microbial-derived recombinant pectinase can replace chemical pectin removal agents, reducing the risk of chemical residues in tobacco processing and complying with the development trend of green production [17].

Based on this, the current study used tobacco pulp from the production line of Henan Tobacco Leaf Processing Co., Ltd as the research material. The PG gene isolated from A. *niger* sw06 was cloned into a vector, and a pectinase genetically engineered strain was constructed. Through screening for high-yield strains, the optimized strain demonstrated effective pectin degradation in tobacco leaves, ultimately contributing to enhanced tobacco leaf quality.

## 2 Materials and methods

### 2.1 Fungal strain

The *Aspergillus niger strain* sw06 was isolated and screened from tobacco field soil in Yunnan Province. This strain was identified by Shanghai Sanen Biotechnology Co., Ltd (Shanghai, China) using a 575 bp sequence length, and its sequence was found to be completely identical (100% homology) to that of the *Aspergillus niger strain* KHSMF44, thereby confirming it as *Aspergillus niger*. The *Pichia pastoris strain* X33 was provided by Shanghai Health Industry Co., Ltd. The identification of this Fugal strain was primarily based on the internal transcribed spacer (ITS) region of the ribosomal RNA gene, specifically including partial sequences of ITS1, the complete sequence of the 5.8S rRNA gene, and the complete sequence of ITS2, combined with partial sequences of the 28S rRNA gene. The gene sequence of this fungus has been submitted to NCBI, with the accession number KP159438.1 GI:76881007.

### 2.2 Kits, enzymes, vectors, and reagents

Total RNA extraction was performed using the Total RNA Extractor Kit from Shanghai Sangon Biotech Co., Ltd (Shanghai Sangon Biotech); the cDNA synthesis kit, primers, and Pfu high-temperature polymerase were also purchased from the same company; the cloning vector pGAPZaA was provided by Shanghai Sangon Biotech Co., Ltd. Culture media included YPD-Genomycin agar plates for strain screening, BMGY and BMMY media for bacterial culture and induction, and super optimal broth (SOB) medium for preparing competent cells. Additionally, the reagents used in the experiment included sorbitol, chloroform, isopropanol, and 75% ethanol (prepared with RNAse-free ddH₂O), while the substrate polygalacturonic acid was purchased from Sigma.

### 2.3 Instrument

Vertical high-speed centrifuge (Japan HITACHI), conversion instrument (Bio-Rad), analysis balance, pH meter (METTER TOLEDO), constant temperature water bath (Jintan Huafeng), centrifuge (Shanghai Anting), 722 spectrophotometer (Shanghai Yidian), magnetic agitator (Gongyi Yuhua), and electroporator (Thermofisher) were used for this research.

### 2.4 Extraction of total RNA from *Aspergillus niger* sw06

The mycelium of *Aspergillus Niger* cultured overnight was fully ground in liquid nitrogen until it was powdered, and RNA was extracted by Trizol method.

#### 2.4.1 RNA extraction

Grind overnight-cultured *Aspergillus niger* mycelium thoroughly in liquid nitrogen until it becomes powder, then extract RNA using the Trizol method. 50–100 mg of mycelium powder was placed in a 1.5 mL centrifuge tube and homogenized with 1 mL of TRIzol reagent by thorough mixing, followed by 5 min incubation at room temperature. Subsequently, 0.2 mL of chloroform was added, the mixture was vortexed vigorously for 15 s and allowed to stand for 2 min before centrifugation at 12,000 g for 15 min at 4°C to separate phases. The aqueous supernatant was transferred to a new tube, mixed with 0.5 mL of isopropanol by gentle inversion, and incubated at room temperature for 10 min to precipitate RNA. After centrifugation at 12,000 g for 10 min at 4°C, the supernatant was discarded and the RNA pellet was washed with 1 mL of 75% ethanol, followed by centrifugation at 7,500 g for 5 min at 4°C. Finally, the air-dried RNA pellet was dissolved in an appropriate amount of DEPC-treated H₂O (promote dissolution at 65°C for 10–15 min).

#### 2.4.2 RNA detection

RNA has its maximum absorption peak at 260 nm. Therefore, RNA concentration can be determined by spectrophotometry at 260 nm, with an optical density (OD) value of 1 corresponding to approximately 40 μg/mL of single-stranded RNA [18]. When performing spectrophotometric quantification using a 1 cm pathlength cuvette, the DNA sample should be diluted n-fold with ddH_2_O, using ddH_2_O as the blank control. The concentration of the sample before dilution can be calculated based on the OD_260_ value read at this point.

\[\text{RNA}(\text{mg}/\text{mL})=40\times {\text{OD}}_{260}\hspace{.5em}\text{reading}\times \text{dilution factor}(n)/\mathrm{1,000}.]\]

The OD_260_/OD_280_ ratio for pure RNA is 2.0, so the purity of RNA can be estimated based on the OD_260_/OD_280_ ratio. A low ratio indicates the presence of residual proteins; a high ratio suggests RNA degradation.

### 2.5 Construction of expression vectors

Synthesis of the first strand cDNA. According to the measured concentration of Total RNA of samples, 1 µL solution was accurately removed for reverse transcription to obtain 20 μL cDNA (RT product). The following reagents were added to the RNase-free tubes: Oligo-(dT) (0.5 µg/μL), 1 μL; Total RNA, X (1 µg) μL; RNase-free H_2_O, constant volume to 12 μL.

The procedure was performed according to the operating instructions of the first cDNA synthesis kit (Dalian Baogong): centrifuge for 5 s after mixing, bathe at 65°C for 5 min, then cool and centrifuge for 5 s. The tube in ice bath was added with the following reagents: 5× Reaction Bμffer, 4 μL; RNase Inhibitor (20 μ/μL), 1 μL; dNTP Mix (10 mmol/L), 2 μL; AMV RT (10 μ/μL), 2 μL. The above reagents were mixed and centrifuged for 5 s. RT-PCR was carried out at 42°C for 60 min and then heating at 85°C for 5 min, and the obtained reverse transcription products were stored at −20°C for later use.

Amplification of target cDNA. Two specific primers were designed according to the start codon and stop codon of galacturonase gene from *A. niger* sw06, the upstream primer: A7488MJ1FN (GACGAATTCCATCATCATCATCATCATGCTCCTTCTCGCGTCTCCGA) and downstream primer: A7488MJ1RN (GACTGCGGCCGCTTAGCAAGAAGCACCGGAAGG). Using the gene sequence of strain sw06 as the template for PCR amplification, the product segment was about 1,079 bp. The amplification system: the total volume of PCR was 50 μL, the first strand cDNA as the template, and the following reagent were added into the sterile microcentrifuge tube [19]: A7488MJ1FN, 2 μL; A7488MJ1RN, 2 μL; Template, 1 μL; dNTP, 1 μL (25 mM each); 10 × pfμ Buffer, 5 μL; Pfμ, 0.4 μL (5 µ/μL), then replenish with ddH_2_O to 50 μL. The procedure reacts at 95°C for 3 min, (95°C 30 s, 50°C 30 s, 72°C 70 s), with 22 cycles, at 72°C for 8 min and then the reaction products were stored at 4°C.

After PCR, 15 μL of amplified products were identified by agarose gel electrophoresis and the obtained images were analyzed. The DNA fragments were recovered according to the operation instructions on the centrifuge column agarose gel DNA recovery kit (UNIQ-10, Shanghai Sheng gong), and purified according to the instructions of the PCR purification kit.

Transformation of link vectors and screening of positive clones. The receptive cells were prepared by selecting strain X33 from plate YPD and inoculating it into medium YPD (20 mL). The recovered PCR product was mixed with pμC57 vector, and the reaction condition was at 16°C for 2 h, and then stored at 4°C [20]. The reaction system: PG, 4 μL; pμC57 Vector, 1 μL; Solution Ⅰ, 1 μL.

All the conjugated products (10 μL) were added into *Pichia pastoris* X33 receptive cells (100 μL) and gently mixed in ice bath for 30 min. After heating shock treatment at 42°C for 90 s, standing tube was cooled in ice bath for 5 min. Then, 900 μL SOB medium was added, which was oscillating incubated with 150 rpm at 37°C for 1 h.

One hundred μL positive clone DH5α was removed and coated, then incubated at 37°C for 12 h to form a single colony. Restriction endonuclease system: plasmid, 37 μL; EcoRI, 1 μL (10 μ/μL); NotI, 1 μL (10 μ/μL); 10× Bμffer BamHI, 10 μL. The enzyme digestion system of the new vector was as follows: pPICZαA, 1 μg; EcoRI, 1 μL (10 μL/μL); NotI, 1 μL (10 μL/μL); 10× Bμffer BamHI, 10 μL. The enzyme digestion of new vector was performed from the correctly sequenced plasmid, reaction sustained 3 h at 37℃. Then, the target gene was bound to the treated new vector. The binding systems of target gene and new vector were as follows: enzyme cut fragment, 10 μL (100 ng); enzyme digestion vector, 3 μL (100 ng); 10× T4 DNAligase Bμffer, 2 μL; T4 DNAligase, 1 μL (5 μ/μL); replenish with ddH_2_O to 20 μL.

According to the method described by Yu et al. [20], 20 μL of linearized monocopy plasmid was mixed with 80 μL of X33 competent cells. The mixture was then spread onto selective plates containing 0.5 mg/mL zeocin (200 μL per plate). Positive clones grown on the selection plates were subsequently isolated, and their plasmids were extracted for electrophoretic verification.

### 2.6 Measured enzyme activity expression and growth curve of X33/pPICZαA-PG

The activity of enzymes was measured according to the method established by Miller [21]. The protocol is as follows:

0.4 mL of 1% pectin solution and 1.0 mL of 0.04 mol/L Na_2_HPO_4_-0.02 mol/L citric acid buffer (pH 5.0) was pre-incubated in a water bath at 45°C for 5 min. Subsequently, 0.1 mL of appropriately diluted enzyme solution (0.5 mg/mL) was added to initiate the reaction, which proceeded at 45°C for 30 min (with heat-inactivated enzyme solution serving as the blank control). The reaction was terminated by adding 3.0 mL of DNS (1% w/v) reagent, followed by boiling for 5 min to develop color. After cooling to room temperature, the reaction mixture was diluted to a final volume of 15 mL. The absorbance was measured at 540 nm wavelength by spectrophotometer, and galacturonic acid was quantified.

Enzyme activity calculation formula:

\[U=({C}_{\text{Sample}}-{C}_{\text{Control}})\hspace{.25em}\times N\times \mathrm{1,000}\times \hspace{.25em}({V}_{\text{T}}/{V}_{\text{E}})/t,]\]

where *N* is the dilution ratio of enzyme solution, *V*_T_ is the total volume of reaction, *V*_E_ is the total volume of enzyme fluid, and *t* is the time.

A standard curve was prepared using a 1.0 mg/mL galacturonic acid solution. 0, 0.2, 0.4, 0.6, 0.8, and 1.0 mL of the standard solution were transferred into separate test tubes, and the volume in each tube was adjusted to 1.0 mL with deionized water. Subsequently, 3.0 mL of DNS reagent was added to each tube, followed by thorough mixing. The mixtures were then incubated in a boiling water bath for 7 min. After cooling to room temperature, the final volume was brought to 15 mL. Absorbance was measured at 540 nm using a UV spectrophotometer.

X33/pPICZαA-PG constructed with YPD liquid culture was inoculated into 120 mL BMGY medium at 4% inoculated volume, fermented for 24 h, inoculated into 3 L BMMY medium for culture, and then induced to express for 54 h. Methanol was added every 12 h at the induced concentration of 0.5%. Samples were taken every 6 h and the dry weight, pH and enzyme activity of the bacteria were photographed and measured at a fermentation temperature of 28℃ (220 rpm) to determine the optimum time for enzyme production. The supernatant after centrifugation was the PG solution expressed by X33, and the enzyme activity was measured. SDS-PAGE detection was performed using sample with volume 10 μL.

### 2.7 Purification and identification of recombinant proteins

The induced expression system of the step of measured enzyme activity expression and growth curve of X33/pPICZαA-PG was used for culture, and the protein expression level was about 0.2 mg/mL. The supernatant after centrifugation was collected and dialyzed into 50 mM Tris, 300 mM NaCl, and pH = 8.0 buffer solution. The supernatant was dialyzed at 4℃ for 12 h, centrifuged at 12,000 rpm for 30 min, and then passed through the membrane for nickel agarose affinity chromatography.

Ten mL Ni-NTA was added into column, then the column was cleaned and balanced at a flow rate of 5 mL/min with binding buffer (50 mM Tris, 300 mM NaCl, 0.1%TritonX-100, pH = 8.0). The volume of binding buffer was ten times that of the column volume. After the supernatant and column packing were mixed evenly, the sample was added into the column at a flow rate of 2 mL/min to collect penetrating liquid. Wash Buffer (50 mM Tris, 300 mM NaCl, 10/20 mM imidazole, pH = 8.0) was used for eluting and collection at a flow rate of 5 mL/min. Elution collection was then performed with Elution Buffer (50 mM Tris, 300 mM NaCl, 500 mM imidazole, pH = 8.0) at 2 mL/min.

Following SDS-PAGE analysis, the 500 mM imidazole eluent was dialyzed against a dialysis buffer (20 mM Tris, pH 7.4), filtered, aliquoted, and stored at –80°C for further use.

The purified protein was determined by a non-interfering protein concentration assay kit (Shanghai Sangon Biotech SK3071). The concentration of BSA was 2 mg/mL and the volume of recombinant protein was 4 μL.

### 2.8 Application of tobacco sheet preparation

The recombinant yeast was induced and cultured by BMMY medium, centrifuged after fermentation for 36 h, and the supernatant liquid was frozen and dried to produce crude enzyme powder. Tobacco stem (4.34% pectin content) and tobacco fines (4.59% pectin content) were evenly mixed at a 5:4 mass ratio. The mixture was combined with water at a solid-to-liquid ratio of 9:20 (w/v) and thoroughly blended to produce tobacco pulp. Mechanical extrusion was employed to separate the pulp into an extract and filter residue. The residue was subsequently repulped, formed into paper sheets, and dried to yield a substrate with a final moisture content of 10% (w/w). The concentrate solution was reduced to 1/8 of the original volume and coated onto the prepared substrate at a 1:4 (w/w) concentrate-to-substrate ratio. The coated substrate was then dried to produce tobacco sheets with a final moisture content of 12% (w/w), yielding the reconstituted tobacco product. The pectinase was added to tobacco pulp at the dosage of 0.4, 0.6, and 0.8%, respectively, with a temperature of 40°C for 3 h. The pectin content in paper-making reconstituted tobacco was identified by carbazole colorimetry from experimental samples [22]. R: Degradation rate of pectin, Ca: Pectin content of concentrated solution before enzymatic hydrolysis, and Cb: Pectin content of concentrated solution after enzymatic hydrolysis.

Recycled tobacco leaves are cut into small pieces and processed into individual tobacco leaf pieces. These are then further processed into reconstituted tobacco strands. The tobacco leaf pieces are stored in a constant temperature and humidity chamber (relative humidity: (60 ± 5)%, temperature: (22 ± 2)°C) for 48 h before use, and are manually rolled into cigarettes. The experimental group consisted of cigarettes treated with enzymatic hydrolysis, while the control group consisted of cigarettes treated with inactivated enzyme solution. The samples were rolled according to the standard of 0.80 g ± 0.01 g per cigarette, in compliance with national standard [23], and balanced for 24 h in a constant temperature and humidity chamber at (22 ± 2)°C and (60 ± 5)% relative humidity. According to the current national standard for the evaluation of finished cigarettes GB5606.4-2005, the quality of cigarettes before and after enzymatic hydrolysis was assessed and evaluated from three aspects: aroma characteristics, smoke characteristics, and taste characteristics.

## 3 Results and discussion

### 3.1 Total RNA quality identification and cDNA amplification

**Figure 1:**
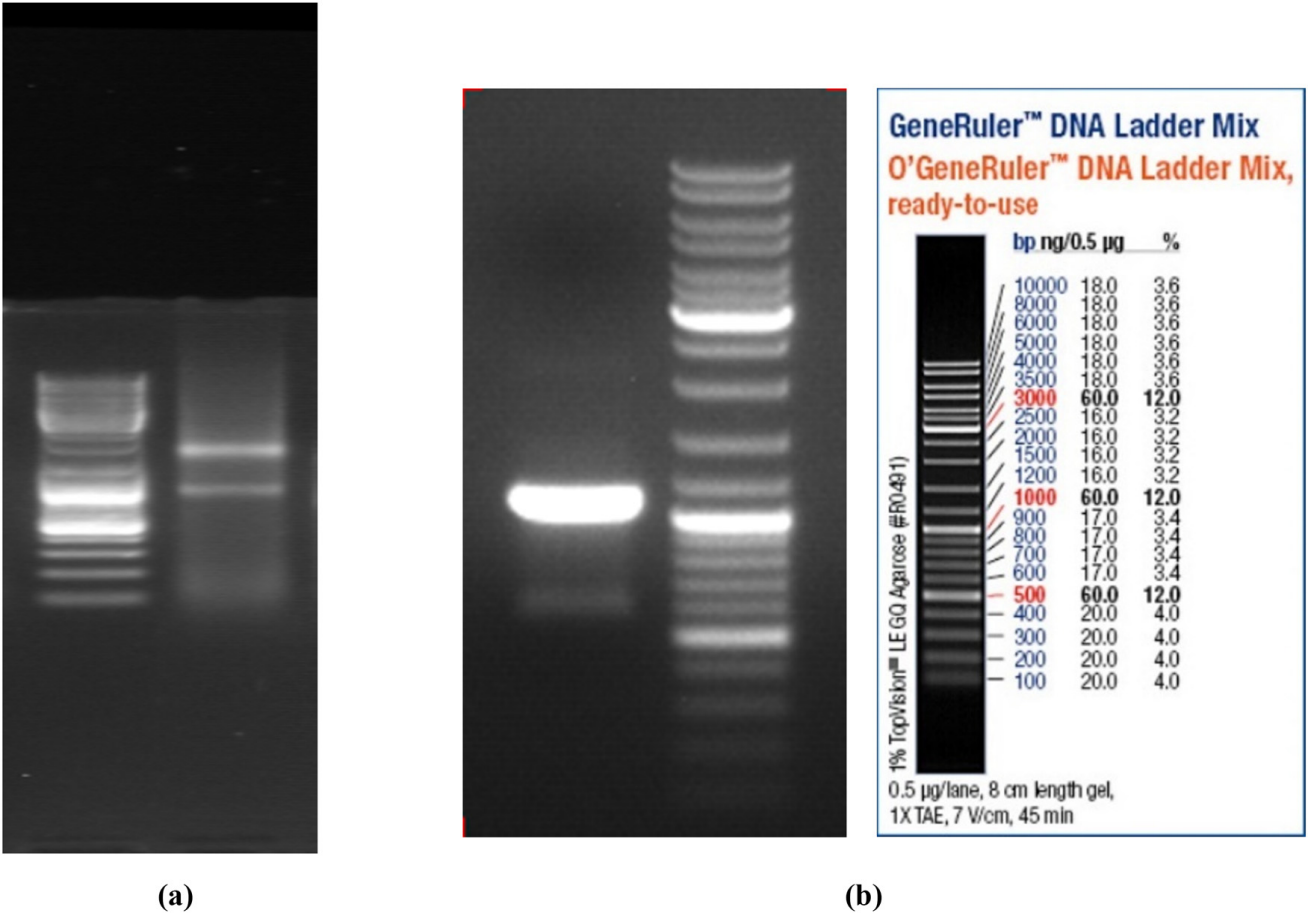
Total RNA detection of *A. niger* (a) and electrophoresis of PCR amplification products of the cDNA (b).

**Figure 2:**
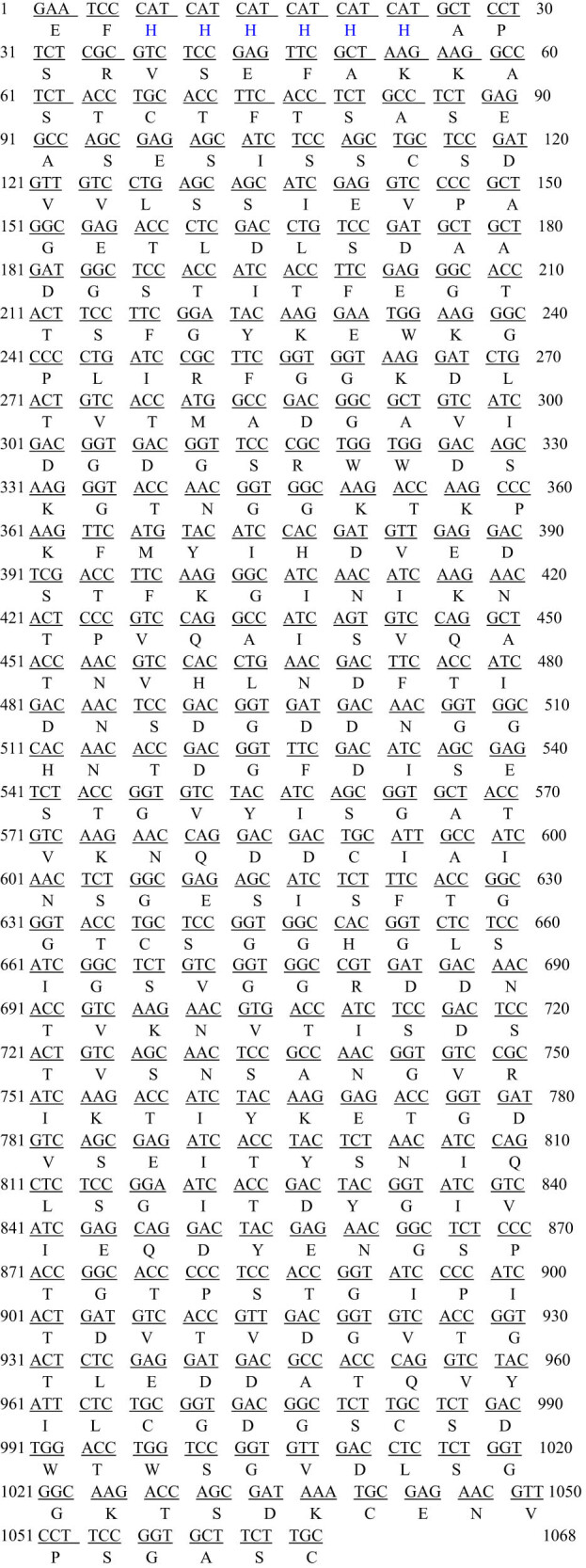
The gene sequence and amino acid sequence of PG from *A. niger* sw06.

**Figure 3:**
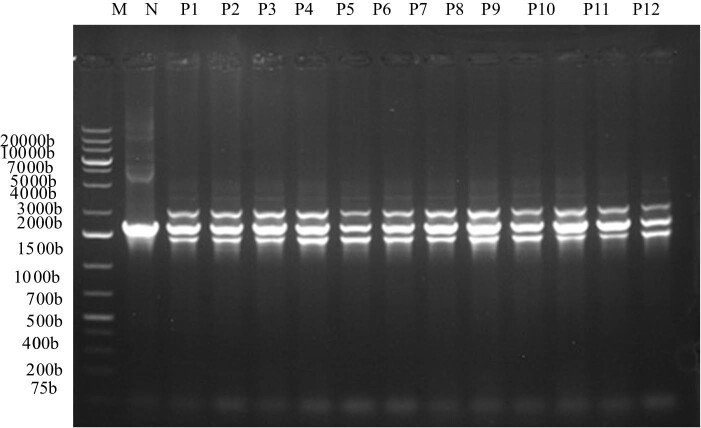
Electrophoresis of the positive clones carrying expression vector X33/pPICZαA-PG by PCR amplification. Annotation: Lane M: DNA molecular weight standard; Lane N: Negative control; Lanes P1–P12: PCR products of positive clones.

Total RNA extracted from *A. niger* sw06 was identified by agarose-gel electrophoresis with 1.5% mass fraction, and the results are shown in Figure 1. It can be seen from Figure 1 that the high-quality RNA was suitable for subsequent experiments. After the first strand cDNA of *A. niger* sw06 was synthesized by reverse transcription, the *A. niger* PG gene was amplified by PCR, and the electrophoretic diagram is shown in Figure 2. The sequencing analysis showed that the total length of the gene was 1,079 bp. Subsequent sequence analysis using the NCBI database revealed a 97% similarity between the obtained nucleotide sequence and that of *Aspergillus niger*. Based on this high sequence homology, the nucleotide sequence was identified as the PG from *A. niger* sw06. The total gene sequence of PG of *A. niger* and its encoded amino acid sequence are shown in Figure 3. Then, the expression vector pGAPZaA-PG was constructed by homologous recombination. The recombinant vector pPICZαA-PG, which contains the 1,079 bp PG gene insert, has a molecular weight of approximately 4.7 kb, which is approximately 1.1 kb larger than that of the empty vector pPICZαA (approximately 3.6 kb), consistent with theoretical calculations.

### 3.2 PCR identification of positive cloning vector

**Figure 4:**
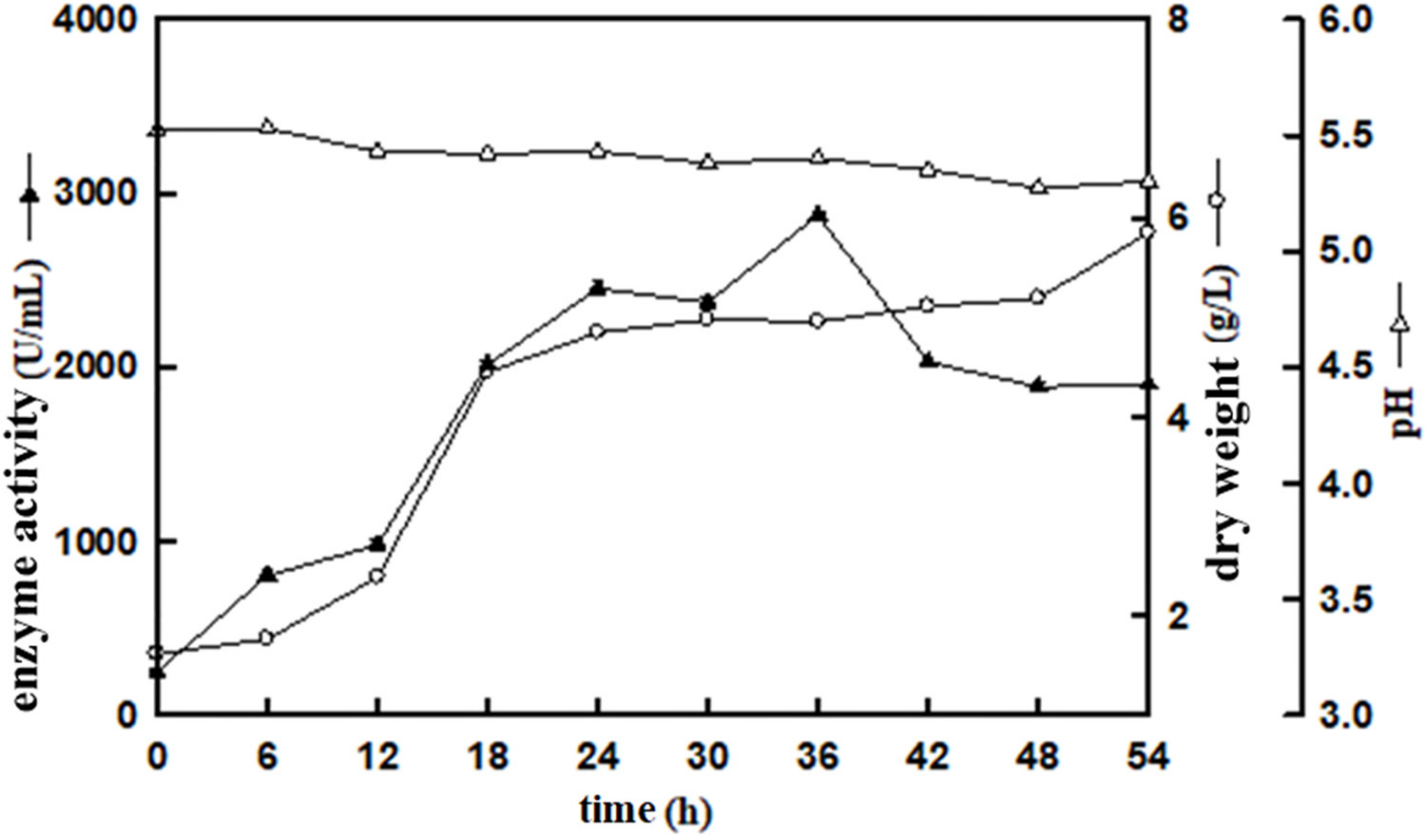
The growth curve of positive clone strain X33/pPICZαA-PG. Annotation: 
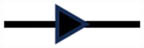
 Enzyme activity (U/mL), 
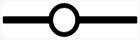
 Dry weighrt(g/L), 
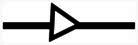
 pH.

The electrophoresis diagram in Figure 4 revealed that two bands were amplified in the transformed sub-genome. The blue arrow in the figure showed the AOX gene in the genome, about 2,200 bp. The other was a vector containing the target gene (about 1,079 bp), about 1,608 bp. Yeast genome (labor number SK8228) was extracted and annealed at 55℃, 30 cycles, using carrier universal primer AOX. The X33/pPICZαA-PG plasmid served as the positive control template. The recombinant strains generated via transformation—designated as X33/pPICZαA-PG < P1–P12 >, corresponding to 12 individual transformant clones—were used as test samples for PCR verification.

### 3.3 Enzyme activity expression and growth cycle identification of positive clone X33/pPICZαA-PG

**Figure 5:**
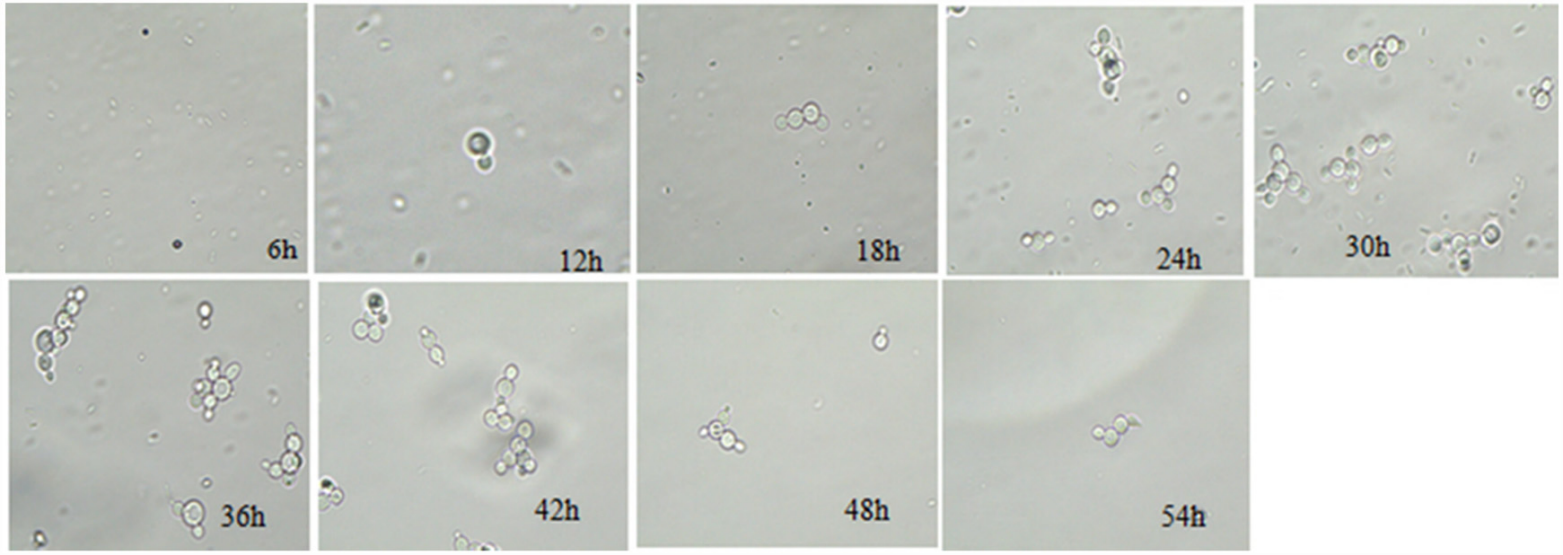
The microscopic structure of positive clone strain X33/pPICZαA-PG.

**Figure 6:**
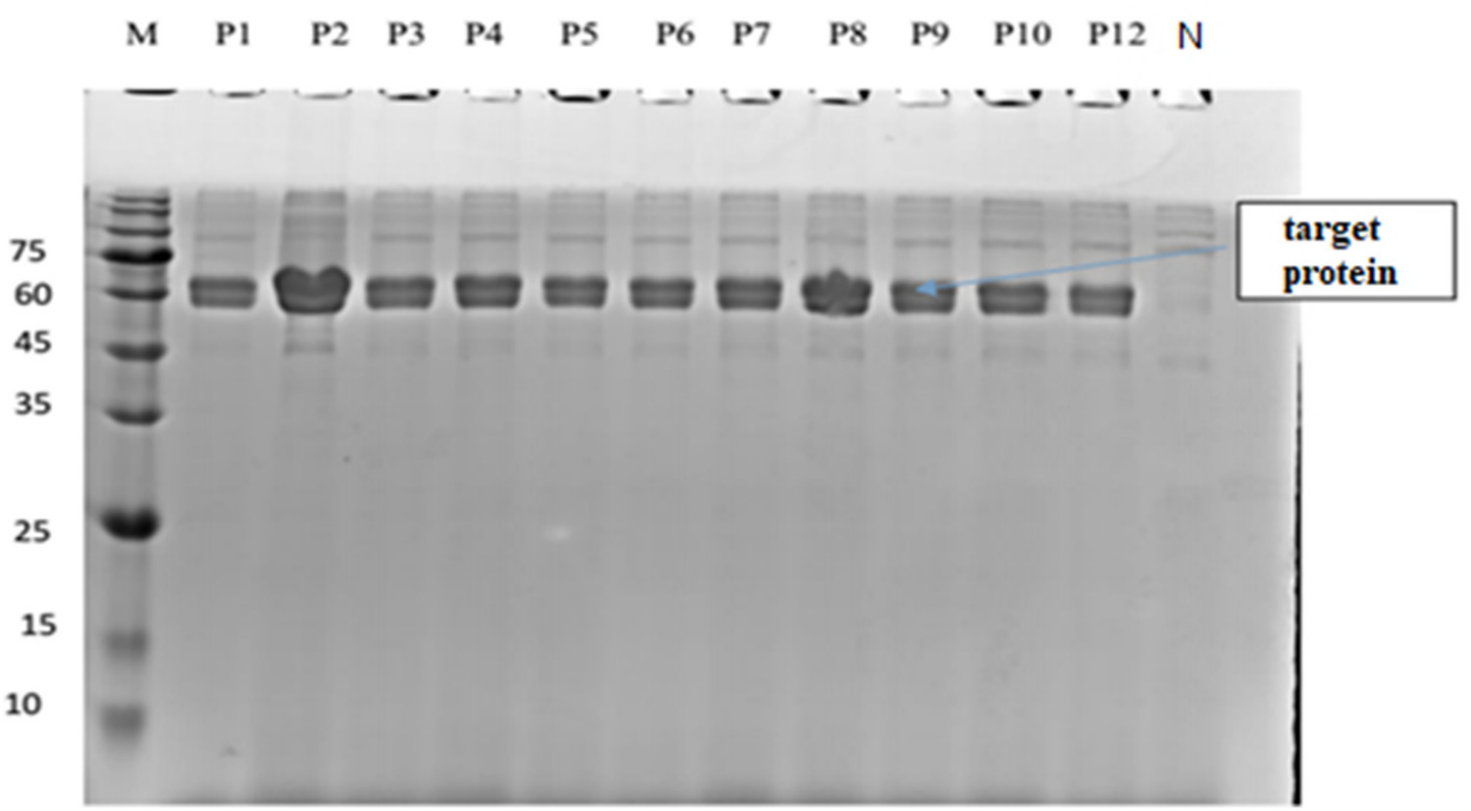
The SDS-PAGE electrophoresis of the target protein from positive clone strain X33/pPICZαA-PG. Annotation: M, Marker, 75 60 45 35 25 15 10 kDa; N, Negative, X33/pPICZαA; P1-P12, Positive, X33/pPICZαA-PG.

The positive clone X33/pPICZαA-PG was cultured in fermenter for 54 h, and the growth curve of X33/pPICZαA-PG was obtained by measuring the changes in dry weight, pH, and enzyme activity. Figure 5 showed that the pH value remained stable between 5.3 and 5.5 during the culture period. The enzyme activity of PG increased gradually in the first 36 h, and then decreased after reaching the peak at 36 h. The maximum PG activity reached 2872.91 U/mL at 36 h, indicating that the optimal culture time was 36 h. As shown in Figure 6, microscopic observation of the positive clone strain X33/pPICZαA-PG revealed that *Pichia pastoris* began budding and differentiation at 12 h. In conjunction with Figure 5, the mycelial dry weight increased rapidly between 12 and 18 h, suggesting vigorous cellular growth during this period.

### 3.4 Expression of target proteins, purification of recombinant proteins, and determination of protein concentration after purification

**Figure 7:**
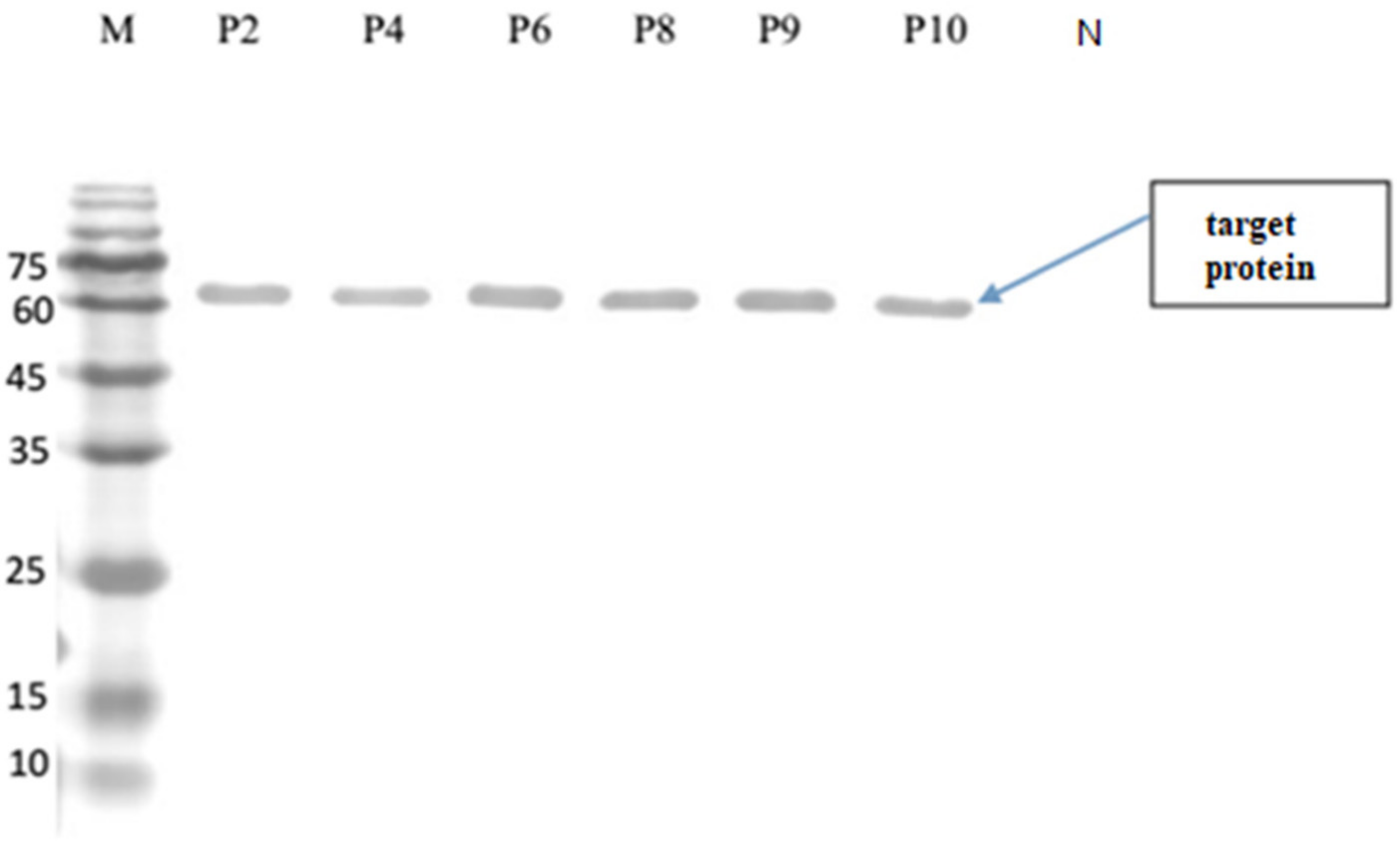
Western blotting identification of the target protein from positive clones strain X33/pPICZαA-PG. Annotation: M, Marker, 75 60 45 35 25 15 10 kDa; N, Negative, X33/pPICZαA; P2, P4, P6, P8, P9, P10, positive, X33/pPICZαA-PG.

**Figure 8:**
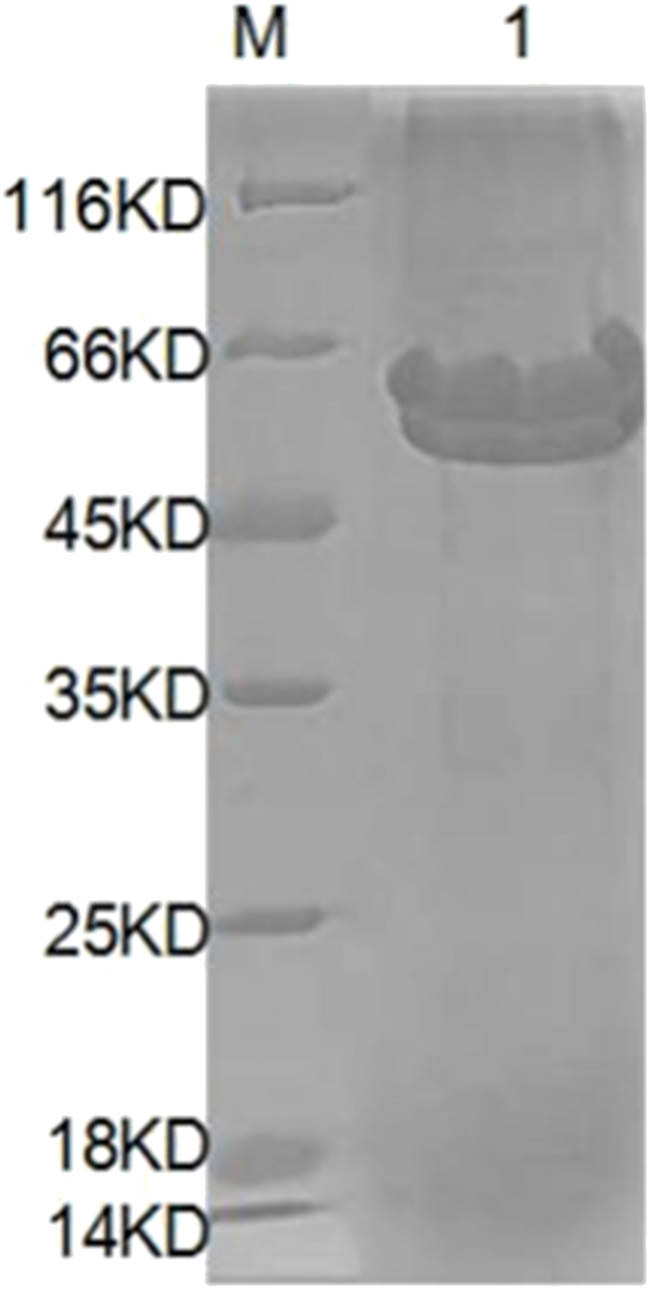
The pure detection of recombinant protein. Annotation: M, protein marker; 1, purified target protein.

SDS-PAGE electrophoresis of the target protein in Figure 7 showed that the molecular weight of the protein was about 60 kDa, which was close to the theoretical analysis of the protein segment of 64 kDa. As can be seen from the western blot identification diagram in Figure 8, the molecular weight of the protein was about 60 kDa, which was close to the theoretical analysis of the protein segment of 64 kDa.

As shown in Figure 8, the molecular weight of recombinant protein was 60 kDa, and it had a high concentration that may have contributed to some degree of diffusion during electrophoresis.

The standard curve of BSA was *y* = ‒0.0078*x* + 0.9486, and *R* ^2^ = 0.9916. After purification of recombinant protein, the protein concentration was 8.1 μg/μL identified by SK3071 kit, indicating a high expression.

Compared with previous studies, Ibrahim et al. [24] expressed the PehA gene encoding PG carotovorum in Escherichia coli, reporting a molecular weight of 41.5 kDa; Liu et al. [25] expressed the cohesive PG (pehA gene) in Pichia pastoris, with a molecular weight of 40 kDa, both of which are lower than the molecular weight of the recombinant protein in this study. This difference may be attributed to variations in the amino acid sequences of PG from different sources. The recombinant protein produced in this study through genetic engineering demonstrated a molecular weight and functional properties consistent with those of its natural counterpart, confirming successful cloning and expression of the target gene [26].

### 3.5 Laboratory experiment and sensory evaluation results

Table 1 revealed that tobacco pulp, concentrated solution, and extract solution had a high content of water, which were suitable for enzyme application. However, the content of pectin in concentrated solution and extract solution was very low, so tobacco pulp was selected as the enzyme addition point. The tobacco pulp had a water content of 96.83%. After enzymatic hydrolysis with recombinant PG at concentrations of 0.4, 0.6, and 0.8%, followed by sheet forming, the pectin content decreased (Table 2). The 0.8% enzyme dosage showed the most effective degradation, reducing the pectin content from 3.65 to 3.01%, achieving a degradation rate of 17.53%. This result confirms the degradation activity of the recombinant enzyme on tobacco pectin, which is consistent with the high expression characteristics of the recombinant enzyme (8.1 μg/μL) in previous fermentation experiments, indicating that the enzyme preparation produced by the engineered strain X33/pPICZαA-PG has practical application potential.

**Table 1:** Pectin content and moisture content of each sample

	Tobacco stem	Tobacco fines	Tobacco pulp	Extract	Concentrated solution	Substrate	Product
Moisture content %	10.1	11.6	96.83	88.25	55.35	9.45	9.9
Dry weight pectin content %	4.34	4.59	3.65	0.09	0.60	4.94	5.54

**Table 2:** Pectin content prior to and after enzyme degradation in tobacco pulp

Tobacco pulp	Pectin content				Degradation rate		
	Pectin content	0.4% after enzymatic hydrolysis	0.6% after enzymatic hydrolysis	0.8% after enzymatic hydrolysis	0.4%	0.6%	0.8%
Pectin content	3.65	3.44	3.19	3.01	5.75	12.60	17.53

Compared with existing studies, although pectinase derived from Penicillium can achieve a higher degradation rate of 28.6% [8], the recombinant enzyme in this study was obtained through a Pichia pastoris expression system, which has advantages such as high product stability and a simple purification process, making it more suitable for the safety and controllability requirements of enzyme preparations in industrial production. Additionally, a degradation rate of 17.53% is sufficient to effectively reduce pectin residues in tobacco pulp, providing a material basis for flavor improvement.

Sensory evaluation results (Table 3) showed that different enzyme addition levels had varying effects on flavor. At 0.4% enzyme addition, the treated tobacco exhibited enhanced aroma and reduced impurities, though with a somewhat weak aftertaste. The 0.6% enzyme treatment produced finer smoke with pleasant taste characteristics, albeit with slightly low concentration. Notably, the 0.8% enzyme treatment made the smoke smooth, taste strong, and aftertaste improved. However, it might have led to excessive pectin degradation, potentially causing an imbalance in flavor compound release.

**Table 3:** Results of sensory evaluation

Sample	Fragrance	Taste	Miscellaneous gas	Burning sensation	Dry sensation	Vestigial
Tobacco pulp	6.00	6.00	6.00	6.00	6.00	6.00
0.4%	6.25	6.50	6.50	6.00	6.00	5.75
0.6%	6.00	6.00	5.75	6.00	6.50	6.25
0.8%	6.00	6.50	6.00	5.75	6.75	7.00

This phenomenon can be further explained by referring to the research by Fan et al. [9], who demonstrated that pectinase treatment promotes the accumulation of volatile aromatic components in tobacco pulp, such as carotenoid degradation products and phenylalanine metabolites. Moderate degradation (such as 0.4% enzyme addition rate) can optimize the release of flavor compounds by improving cellular structure, while excessive enzyme levels may disrupt the matrix’s ability to retain flavor components, leading to reduced flavor harmony. Therefore, it is recommended to add 0.4% enzyme in tobacco pulp.

## 4 Conclusion

This study cloned a 1,079 bp PG gene from the *Aspergillus niger* sw06 strain, which shares 97% sequence similarity with known A*spergillus niger* PG genes. A recombinant vector pPICZαA-PG was constructed using EcoRⅠ and NotⅠ, and transformed into *Pichia pastoris* X33 to obtain the engineered strain. Sequencing confirmed the successful integration of the target gene (1,608 bp in the transformed genome). The crude enzyme activity reached a peak of 2,872.91 U/mL after 36 h of cultivation in the recombinant yeast. In tobacco pulp applications, 0.8% recombinant PG achieved a pectin degradation rate of 17.53%; the most significant improvement in tobacco flavor was observed at a 0.4% addition rate. This study provides genes, strains, and optimized parameters related to efficient enzyme preparations for pectin degradation in tobacco processing.
